# Supplementary material for: A highly efficient and accurate method of detecting and subtyping Influenza A pdm H1N1 and H3N2 viruses with newly emerging mutations in the matrix gene in Eastern Taiwan
Source: PLoS One. 2023 Mar 23;18(3):e0283074. doi: 10.1371/journal.pone.0283074 (PMC10035893; doi:10.1371/journal.pone.0283074)
Supplement: S1 Table — (DOCX) [file pone.0283074.s001.docx]

## S1 Table. C_T_ values of Influenza A pdm H1N1 detected from representative clinical samples and viral isolations by the two real-time RT-PCR assays.

| Specimen no. | *C_T_* values from the Indicated assay | |  |  |
| --- | --- | --- | --- | --- |
|  | MAF/MAR | InfAF/InfAR | HA clade (A pdm H1N1) | Collection date |
| 486268 | 23.44 | 20.73 | NA | Jan/2011 |
| 486293 | 30.73 | 27.53 | 12 | Jan/2011 |
| 486009 | 28.05 | 25.37 | 10 | Jan/2011 |
| 438700 | 30.40 | 28.21 | 12 | Jan/2011 |
| 438799 | 22.31 | 19.98 | 12 | Feb/2011 |
| 438820 | 31.07 | 28.35 | 10 | Feb/2011 |
| 438836 | 18.32 | 16.72 | 12 | Feb/2011 |
| 438877 | 19.32 | 17.65 | 12 | Feb/2011 |
| 438859 | 17.10 | 15.22 | 12 | Feb/2011 |
| 866896 | 25.50 | 23.56 | 10 | Jun/2011 |
| 277957 | 24.80 | 22.65 | 12.3 (new) | Jan/2012 |
| 739148 | 22.91 | 20.66 | 12.3 (new) | Feb/2012 |
| 739158 | 33.41 | 29.83 | 12.3 (new) | Feb/2012 |
| 739233 | 30.31 | 27.00 | 12.3 (new) | Mar/2012 |
| 739470 | 27.73 | 25.33 | 12.3 (new) | May/2012 |
| 739540 | 23.35 | 20.67 | 12.3 (new) | May/2012 |
| 739610 | 26.59 | 24.04 | 12.3 (new) | Jun/2012 |
| 739614 | 33.89 | 30.12 | 12.3 (new) | Jun/2012 |
| 444806 | 23.87 | 19.14 | 12.3 (new) | Aug/2012 |
| 739969 | 27.52 | 23.39 | 12.3 (new) | Sep/2012 |
| 123500 | 28.85 | 26.37 | 12.2 | Mar/2013 |
| 123555 | 20.80 | 18.89 | 12.1 | Mar/2013 |
| 123660 | 34.03 | 26.86 | 11.1 | Apr/2013 |
| 124000 | 29.06 | 29.94 | 11.1 | Jun/2013 |
| 123722 | 17.61 | 15.92 | 11.1 | Apr/2013 |
| 123809 | 29.10 | 25.40 | 11.1 | May/2013 |
| 142194 | 29.51 | 26.71 | 11.1 | Jul/2013 |
| 142722 | 15.15 | 13.65 | 11.2 (new) | Dec/2013 |
| 142727 | 13.90 | 11.63 | 11.2 (new) | Dec/2013 |
| 142731 | 19.44 | 17.50 | 11.2 (new) | Dec/2013 |
| 142759 | 19.34 | 17.30 | 11.2 (new) | Jan/2014 |
| 142882 | 19.18 | 17.45 | 11.2 (new) | Jan/2014 |
| 142921 | 17.46 | 15.85 | 11.2 (new) | Jan/2014 |
| 142974 | 17.15 | 14.82 | 11.2 (new) | Feb/2014 |
| 146015 | 17.29 | 15.23 | 11.2 (new) | Feb/2014 |
| 146360 | 29.48 | 26.81 | 11.2 (new) | Mar/2014 |
| 838075 | 18.68 | 16.16 | 11.2 (new) | Apr/2014 |
| 838309 | 14.78 | 13.75 | 11.2 (new) | Mar/2014 |
| 838606 | 16.14 | 16.63 | 11.2 (new) | Aug/2014 |
| 380025 | 15.55 | 13.50 | 11.2 (new) | Feb/2015 |
| 380162 | 21.02 | 20.11 | 11.2 (new) | Mar/2015 |
| 380178 | 16.47 | 14.57 | 11.2 (new) | Apr/2015 |
| 380503 | 28.18 | 25.97 | 11.2 (new) | Jul/2015 |
| 380800 | 34.89 | 32.48 | 11.2 (new) | Nov/2015 |

HA, hemagglutinin gene; NA, unavailable.
