# Supplementary material for: A highly efficient and accurate method of detecting and subtyping Influenza A pdm H1N1 and H3N2 viruses with newly emerging mutations in the matrix gene in Eastern Taiwan
Source: PLoS One. 2023 Mar 23;18(3):e0283074. doi: 10.1371/journal.pone.0283074 (PMC10035893; doi:10.1371/journal.pone.0283074)
Supplement: S2 Table — (DOCX) [file pone.0283074.s002.docx]

**S2 Table. The C_T_ value of Influenza A H3N2 detected from representative clinical samples and viral isolations by the two real-time RT-PCR assays.**

| Specimen no. | *C_T_* values from the Indicated assay | |  |  |
| --- | --- | --- | --- | --- |
|  | MAF/MAR | InfAF/InfAR | HA clade A H3N2 | Collection  date |
| 501707 | 31.54 | 21.95 | TW2011-13 | Jan/2011 |
| 501717 | UD | 20.17 | TW2011-13 | Feb/2011 |
| 501818 | 31.37 | 19.45 | TW2011-13 | Feb/2011 |
| 738282 | 28.22 | 21.29 | TW2011-13 | Sep/2011 |
| 500353 | UD | 22.26 | TW2011-13 | Oct/2011 |
| 738438 | UD | 21.23 | TW2011-13 | Oct/2011 |
| 213587 | UD | 19.00 | TW2011-13 | Nov/2011 |
| 500300 | UD | 18.99 | TW2011-13 | Nov/2011 |
| 213591 | UD | 17.17 | TW2011-13 | Nov/2011 |
| 738633 | UD | 18.03 | TW2011-13 | Nov/2011 |
| 739127 | UD | 19.70 | TW2011-13 | Feb/2012 |
| 272976 | UD | 17.26 | TW2011-13 | Mar/2012 |
| 505081 | UD | 21.19 | TW2011-13 | Mar/2012 |
| 277657 | UD | 27.63 | TW2011-13 | Mar/2012 |
| 277674 | UD | 17.62 | TW2011-13 | Apr/2012 |
| 444014 | UD | 20.17 | TW2011-13 | May/2012 |
| 488114 | UD | 16.92 | TW2011-13 | May/2012 |
| 444909 | 28.25 | 20.64 | TW2011-13 | Jun/2012 |
| 444912 | 28.26 | 20.49 | TW2011-13 | Sep/2012 |
| 739928 | UD | 27.64 | TW2011-13 | Sep/2012 |
| 123327 | UD | 32.33 | TW2011-13 | Jan/2013 |
| 123331 | UD | 25.725 | TW2011-13 | Jan/2013 |
| 123367 | 34.03 | 26.86 | TW2011-13 | Feb/2013 |
| 123416 | 29.06 | 19.94 | TW2011-13 | Feb/2013 |
| 123480 | 30.76 | 22.95 | TW2011-13 | Mar/2013 |
| 123661 | 19.05 | 16.92 | TW2011-13 | Apr/2013 |
| 123776 | UD | 31.23 | TW2011-13 | Apr/2013 |
| 142090 | UD | 23.44 | TW2011-13 | Jun/2013 |
| 142699 | UD | 26.38 | TW2014-15 | Dec/2013 |
| 142730 | 33.68 | 24.06 | TW2014-15 | Dec/2013 |
| 142774 | 32.36 | 23.41 | TW2014-15 | Jan/2014 |
| 142891 | 27.54 | 22.76 | TW2014-15 | Jan/2014 |
| 142956 | UD | 23.35 | TW2014-15 | Jan/2014 |
| 146017 | UD | 29.10 | TW2014-15 | Feb/2014 |
| 146346 | 27.02 | 19.36 | TW2014-15 | Mar/2014 |
| 838118 | 29.03 | 19.26 | TW2014-15 | Apr/2014 |
| 8384481 | UD | 24.58 | TW2014-15 | Jul/2014 |
| 838717 | 22.42 | 14.59 | TW2014-15 | Sep/2014 |
| 838796 | UD | 15.02 | TW2014-15 | Oct/2014 |
| 838892 | UD | 14.56 | TW2014-15 | Dec/2014 |
| 838924 | UD | 15.28 | TW2014-15 | Jan/2015 |
| 838545 | 26.10 | 15.35 | TW2014-15 | Jan/2015 |
| 838595 | 25.43 | 14.47 | TW2014-15 | Feb/2015 |
| 380068 | 25.35 | 14.10 | TW2014-15 | Mar/2015 |
| 380218 | UD | 16.77 | TW2014-15 | Apr/2015 |
| 380281 | UD | 17.29 | TW2014-15 | May/2015 |
| 380480 | 29.69 | 17.94 | TW2014-15 | Jun/2015 |
| 380501 | UD | 17.88 | TW2014-15 | Jul/2015 |
| 380595 | UD | 18.06 | TW2014-15 | Aug/2015 |
| 380668 | UD | 31.23 | TW2014-15 | Sep/2015 |

MA and InfA, two types of primers; F/R, forward/reverse; HA, hemagglutinin gene; UD, undetectable.
