# Supplementary material for: A highly efficient and accurate method of detecting and subtyping Influenza A pdm H1N1 and H3N2 viruses with newly emerging mutations in the matrix gene in Eastern Taiwan
Source: PLoS One. 2023 Mar 23;18(3):e0283074. doi: 10.1371/journal.pone.0283074 (PMC10035893; doi:10.1371/journal.pone.0283074)
Supplement: S3 Table — (DOCX) [file pone.0283074.s003.docx]

**S3 Table. Raw data of Table 3**

|  |  | Singleplex assay | | | |  | | Multiplex assay | | | |  | |  |
| --- | --- | --- | --- | --- | --- | --- | --- | --- | --- | --- | --- | --- | --- | --- |
|  |  | C_T_ values | |  | |  | |  | |  | |  | |  |
| **Copies/µL** | Log_10_ Conc. | | MAF/R | MBF/R | | InfAF/R | | MAF/R | | MBF/R | | InfAF/R | |  |
| 100000 | 5 | 19.94 | | 21.87 | | 18.28 | | 21.55 | | 21.78 | | 18.99 | |  |
|  |  | 20.01 | | 22.02 | | 18.15 | | 21.50 | | 21.70 | | 19.03 | |  |
|  |  | 20.58 | | 21.73 | | 18.30 | | 21.41 | | 21.63 | | 18.86 | |  |
|  |  | 20.38 | | 21.82 | | 18.15 | | 22.12 | | 21.74 | | 18.82 | |  |
|  |  | 20.44 | | 22.00 | | 18.04 | | 22.05 | | 21.66 | | 18.86 | |  |
|  |  | 21.14 | | 21.69 | | 18.20 | | 21.96 | | 21.59 | | 18.67 | |  |
|  |  | 19.05 | | 22.38 | | 17.88 | | 20.74 | | 22.35 | | 18.55 | |  |
|  |  | 19.16 | | 22.45 | | 17.75 | | 20.80 | | 22.27 | | 18.54 | |  |
|  |  | 19.74 | | 22.27 | | 18.03 | | 20.60 | | 22.24 | | 18.36 | |  |
|  |  | 20.06 | | 21.55 | | 19.01 | | 21.69 | | 21.50 | | 19.63 | |  |
|  |  | 20.10 | | 21.70 | | 18.74 | | 21.63 | | 21.46 | | 19.54 | |  |
|  |  | 20.73 | | 21.48 | | 18.87 | | 21.53 | | 21.36 | | 19.43 | |  |
|  |  |  | |  | |  | |  | |  | |  | |  |
|  | Mean±SD | 20.11±0.58 | | 21.91±0.3 | | 18.28±0.36 | | 21.47±0.49 | | 21.77±0.32 | | 18.94±0.39 | |  |
|  |  |  | |  | |  | |  | |  | |  | |  |
| 10000 | 4 | 23.36 | | 25.26 | | 21.51 | | 24.99 | | 25.02 | | 22.32 | |  |
|  |  | 23.30 | | 25.24 | | 21.55 | | 25.17 | | 25.16 | | 22.51 | |  |
|  |  | 23.27 | | 25.15 | | 21.53 | | 25.09 | | 25.11 | | 22.44 | |  |
|  |  | 23.88 | | 25.23 | | 21.37 | | 25.43 | | 25.00 | | 22.21 | |  |
|  |  | 23.81 | | 25.21 | | 21.40 | | 25.59 | | 25.13 | | 22.38 | |  |
|  |  | 23.33 | | 25.13 | | 21.40 | | 25.53 | | 25.09 | | 22.30 | |  |
|  |  | 22.55 | | 25.92 | | 21.14 | | 24.21 | | 25.52 | | 22.03 | |  |
|  |  | 22.29 | | 25.84 | | 21.14 | | 24.29 | | 25.71 | | 22.17 | |  |
|  |  | 22.31 | | 25.67 | | 21.19 | | 24.14 | | 25.61 | | 22.07 | |  |
|  |  | 23.47 | | 25.06 | | 22.13 | | 25.09 | | 24.72 | | 22.94 | |  |
|  |  | 23.41 | | 25.06 | | 22.17 | | 25.26 | | 24.97 | | 23.13 | |  |
|  |  | 23.44 | | 24.98 | | 22.12 | | 25.19 | | 24.89 | | 23.11 | |  |
|  |  |  | |  | |  | |  | |  | |  | |  |
|  | Mean±SD | 23.2±0.51 | | 25.31±0.31 | | 21.55±0.36 | | 25.00±0.49 | | 25.16±0.29 | | 22.47±0.37 | |  |
|  |  |  | |  | |  | |  | |  | |  | |  |
| 1000 | 3 | 26.54 | | 28.59 | | 25.16 | | 28.66 | | 28.43 | | 26.13 | |  |
|  |  | 26.64 | | 28.69 | | 24.99 | | 28.64 | | 28.49 | | 26.07 | |  |
|  |  | 27.68 | | 28.52 | | 25.08 | | 28.57 | | 28.52 | | 26.04 | |  |
|  |  | 27.12 | | 28.55 | | 25.06 | | 29.17 | | 28.40 | | 26.04 | |  |
|  |  | 27.15 | | 28.65 | | 24.82 | | 29.17 | | 28.45 | | 25.93 | |  |
|  |  | 28.13 | | 28.48 | | 24.98 | | 29.11 | | 28.48 | | 25.88 | |  |
|  |  | 25.65 | | 29.18 | | 24.81 | | 28.00 | | 29.07 | | 25.72 | |  |
|  |  | 25.87 | | 29.26 | | 24.53 | | 27.88 | | 29.12 | | 25.63 | |  |
|  |  | 27.15 | | 29.16 | | 24.71 | | 27.80 | | 29.12 | | 25.59 | |  |
|  |  | 26.68 | | 28.34 | | 25.71 | | 28.80 | | 28.22 | | 26.68 | |  |
|  |  | 26.80 | | 28.41 | | 25.51 | | 28.79 | | 28.25 | | 26.69 | |  |
|  |  | 27.80 | | 28.25 | | 25.63 | | 28.71 | | 28.30 | | 26.60 | |  |
|  |  |  | |  | |  | |  | |  | |  | |  |
|  | Mean±SD | 26.93±0.71 | | 28.67±0.33 | | 25.08±0.35 | | 28.61±0.46 | | 28.57±0.32 | | 26.08±0.37 | |  |
|  |  |  | |  | |  | |  | |  | |  | |  |
| 100 | 2 | 30.16 | | 32.11 | | 28.50 | | 32.20 | | 32.03 | | 29.67 | |  |
|  |  | 30.20 | | 32.18 | | 28.54 | | 32.45 | | 32.12 | | 30.10 | |  |
|  |  | 30.43 | | 31.98 | | 28.58 | | 32.41 | | 32.03 | | 29.60 | |  |
|  |  | 30.66 | | 32.09 | | 28.38 | | 32.70 | | 32.01 | | 29.51 | |  |
|  |  | 30.71 | | 32.15 | | 28.43 | | 33.06 | | 32.09 | | 30.00 | |  |
|  |  | 31.06 | | 31.93 | | 28.46 | | 32.89 | | 32.01 | | 29.46 | |  |
|  |  | 29.38 | | 32.66 | | 28.18 | | 31.43 | | 32.47 | | 29.24 | |  |
|  |  | 29.41 | | 32.81 | | 28.24 | | 31.57 | | 32.70 | | 29.69 | |  |
|  |  | 29.61 | | 32.46 | | 28.26 | | 31.85 | | 32.48 | | 29.22 | |  |
|  |  | 30.27 | | 31.84 | | 29.09 | | 32.31 | | 31.74 | | 30.32 | |  |
|  |  | 30.31 | | 31.98 | | 29.11 | | 32.59 | | 31.88 | | 30.65 | |  |
|  |  | 30.58 | | 31.66 | | 29.13 | | 32.52 | | 31.76 | | 30.23 | |  |
|  |  |  | |  | |  | |  | |  | |  | |  |
|  | Mean±SD | 30.23±0.51 | | 32.15±0.32 | | 28.58±0.33 | | 32.33±0.48 | | 32.11±0.28 | | 29.81±0.43 | |  |
|  |  |  | |  | |  | |  | |  | |  | |  |
| 10 | 1 | 33.45 | | 35.07 | | 32.01 | | 36.06 | | 35.97 | | 33.45 | |  |
|  |  | 33.40 | | 35.53 | | 32.08 | | 36.11 | | 35.86 | | 33.47 | |  |
|  |  | 33.48 | | 35.38 | | 32.03 | | 36.05 | | 36.06 | | 33.46 | |  |
|  |  | 34.04 | | 35.05 | | 31.86 | | 36.47 | | 35.92 | | 33.33 | |  |
|  |  | 33.98 | | 35.49 | | 31.97 | | 36.56 | | 35.82 | | 33.35 | |  |
|  |  | 34.05 | | 35.35 | | 31.88 | | 36.47 | | 36.04 | | 33.33 | |  |
|  |  | 32.59 | | 35.56 | | 32.59 | | 35.25 | | 36.43 | | 33.12 | |  |
|  |  | 32.57 | | 36.16 | | 31.68 | | 35.28 | | 36.35 | | 33.16 | |  |
|  |  | 32.70 | | 36.05 | | 31.59 | | 35.23 | | 36.50 | | 33.12 | |  |
|  |  | 33.59 | | 34.80 | | 32.49 | | 36.15 | | 35.65 | | 34.08 | |  |
|  |  | 33.52 | | 35.26 | | 32.64 | | 36.21 | | 35.60 | | 34.08 | |  |
|  |  | 33.62 | | 35.16 | | 32.48 | | 36.14 | | 35.80 | | 34.09 | |  |
|  |  |  | |  | |  | |  | |  | |  | |  |
|  | Mean±SD | 33.10±0.51 | | 35.41±0.38 | | 32.11±0.34 | | 36.00±0.46 | | 36.00±0.28 | | 33.50±0.36 | |  |
|  |  |  | |  | |  | |  | |  | |  | |  |
| 1 | 0 | 37.1 | | 37.75 | | 35.48 | | 39.60 | | 37.7 | | 37.3 | |  |
|  |  | 36.63 | | 39.14 | | 35.35 | | 39.27 | | 38.23 | | 37.16 | |  |
| 36.99 | | | | | 39.01 | | 35.28 | | 39.39 | | 38.60 | | 37.20 | |
| 37.56 | | | | | 37.70 | | 35.35 | | 40.15 | | 37.66 | | 37.17 | |
| 37.15 | | | | | 39.11 | | 35.24 | | 39.78 | | 38.20 | | 37.07 | |
| 37.39 | | | | | 38.97 | | 35.15 | | 39.94 | | 38.56 | | 37.10 | |
| 36.23 | | | | | 38.29 | | 35.14 | | 38.80 | | 38.17 | | 36.93 | |
| 35.88 | | | | | 39.74 | | 35.06 | | 38.52 | | 38.84 | | 36.81 | |
| 36.16 | | | | | 39.58 | | 34.91 | | 38.61 | | 39.19 | | 36.88 | |
| 37.20 | | | | | 37.47 | | 36.1 | | 39.76 | | 37.67 | | 38.02 | |
| 36.78 | | | | | 38.90 | | 35.98 | | 39.38 | | 38.05 | | 37.68 | |
| 37.08 | | | | | 38.66 | | 36.00 | | 39.51 | | 38.33 | | 37.73 | |
|  | | | | |  | |  | |  | |  | |  | |
| Mean±SD 36.85±0.50 | | | | | 38.70±0.71 | | 35.42±0.38 | | 39.39±0.50 | | 38.27±0.6 | | 37.25±0.36 | |
|  | | | | |  | |  | |  | |  | |  | |
| R 0.9995 | | | | | 1.0000 | | 0.9999 | | 0.9999 | | 0.9984 | | 0.9999 | |
| Slope -3.3343 | | | | | -3.3637 | | -3.4537 | | -3.6091 | | -3.3874 | | -3.6677 | |
| E 99.4913 | | | | | 98.3145 | | 94.806 | | 89.2716 | | 97.354 | | 87.3718 | |
|  | | | | |  | |  | |  | |  | |  | |
|  | | | | |  | |  | |  | |  | |  | |
|  | | | | |  | |  | |  | |  | |  | |
|  | | | | |  | |  | |  | |  | |  | |
