# Supplementary material for: A highly efficient and accurate method of detecting and subtyping Influenza A pdm H1N1 and H3N2 viruses with newly emerging mutations in the matrix gene in Eastern Taiwan
Source: PLoS One. 2023 Mar 23;18(3):e0283074. doi: 10.1371/journal.pone.0283074 (PMC10035893; doi:10.1371/journal.pone.0283074)
Supplement: Attachment — Submitted filename: Responses to Comments from Editor and Reviewer.pdf [file pone.0283074.s005.pdf]

Responses to Editors and Reviewers (PONE-D-22-10495)

We would like to thank the editor and reviewers for their extensive assessment of our manuscript, and for important and helpful comments and suggestions. We have taken all the remarks into account, in a manner that is described in detail below together with our responses to comments. We have responded to all the editor's and reviewer's comments in a point-by-point fashion and have revised the manuscript accordingly. The revised portions are indicated by "Track Changes". An "unmarked" clean version of revised manuscript has also been provided. We think that, following these suggestions, our manuscript has gained in clarity and hope that the changes made will be considered satisfactory.

One additional remark is that please update our Funding Statement in the online submission, which should read as: "This work was supported by Grants from the Centers for Disease Control, R.O.C (Taiwan). (HZ099103, HZ100102, HZ101060, CH102055, CW103035)."

Responses to Editor:

Comment 1: Please include the following items when submitting your revised manuscript.

Response: We have provided a rebuttal letter that responds to the academic editor and reviewers in a point-by-point fashion. We have uploaded a marked-up copy of our

revised manuscript that highlights changes made to the original version labeled as 'Revised Manuscript with Track Changes'. We have also uploaded an unmarked version without tracked changes labeled as 'Manuscript'.

Comment 2: Please ensure that your manuscript meets PLOS ONE's style requirements, including those for file naming.

Response: We have ensured that our manuscript meets the style requirements of PLOS ONE.

Comment 3: If the need for consent was waived by the ethics committee, please include this information.

Response: We have provided a statement to declare that informed consent was waived according to the institutional guidelines (line 126, unmarked version).

Comment 4: We note that the grant information you provided in the 'Funding Information' and 'Financial Disclosure' sections do not match.

Response: We have corrected this mismatching.

Comment 5: Please remove any funding-related text from the manuscript and let us know how you would like to update your Funding Statement. Please include your amended statements within your cover letter; we will change the online submission form on your behalf.

Response: We have removed the funding-related text from the manuscript. Also, please update our Funding Statement in the online submission, which should read as: "This work was supported by Grants from the Centers for Disease Control, R.O.C (Taiwan). (HZ099103, HZ100102, HZ101060, CH102055, CW103035)."

Responses to Reviewer 1:

Comment 1: The manuscript has to be revised for English language, there is several words used which is giving another meaning and impression like:

(a) line 56 : Influenza A virus strains can "Stably" infect humans... : Stable is not the right word

Response to (a): We have deleted the word “stably” (line 61, unmarked version).

(b) Line 91: Primers and Probes failing to "Adhere" : Bind is much better to use here

Response to (b): Thank you. We have replaced the word by “bind” (line 92, unmarked version).

(c) Line 319: Influenza virus genome segment "Develops" , "evolve" would be the correct word.

Response to (c): Thank you. We have replaced the word by “evolve” (line 329, unmarked version).

(d) the mistakes with the English Language is repeated in several sections of the MS so i would recommend professional English editing before publication.

Response to (d): In response to the recommendation from the reviewer, we have asked help to improve the language of our revised manuscript by a professional English editor (KGSupport). The certificate of this English editing service is provided at the end of this document.

Comment 2: Line :152 , Please give more details regarding your positive and negative controls for both real-time PCR and conventional PCR.

Response: We thank the reviewer for reminding us this important issue. In response to this comment, we have now provided details regarding our positive and negative controls for both real-time PCR and conventional PCR (lines 170-173, unmarked version). The statement reads as: “We used RNA extracted from Influenza A/New Caledonia/20/99(H1N1), Influenza A/Wisconsin/67/05(H3N2) and Influenza B Florida/07/04 as positive controls and sterile water as the negative control in each run.”

Comment 3: You used the Term "RNA sequencing" , this is somehow misleading since you only made sequencing of the viral cDNA and not the RNA.

Response: We thank the reviewer for this suggestion. The misleading term has now been replaced by “viral cDNA sequencing” (lines 193 and 194, unmarked version).

Comment 4: No statistics made, Statistical analysis has to be made to address the significance and compare the sensitivity of the 2 sets of primers and probes for better conclusion.

Response: We thank the reviewer for this excellent suggestion. In response to this comment, we have additionally provided data of analytical sensitivity of the conventional singleplex and our multiplex assays analyzed by standard curves of C_T values. For comparisons of C_T values and Pearson correlation coefficients between the

conventional singleplex and our multiplex assays, we have used independent sample t-tests and no significances were detected. Thus, we have made the following revisions:

(1) This additional set of data is presented as **Table 5** (page 29, unmarked version).

(2) The results of these analyses have been reported in the Result section (lines 248-254 , unmarked version). These statements read as:" Table 5 shows the data of the analytical sensitivity of the multiplex assays developed in this study and the traditional singleplex assays. The C_T values for each standard concentration obtained from the use of MAF/R, MBF/R, and InfAF/R in the multiplex assays did not significantly differ from those in the singleplex assays. Further analyses of standard curves of C_T values revealed that the correlation coefficients, slopes, and amplification efficiencies of these two types of assays had no significant differences."

(3) The results of these analyses have been reported in the abstract (lines 52-53, unmarked version) and the statement reads as: "The analytical sensitivity of this multiplex RT-PCR assay was as good as that of the conventional singleplex assay."

(4) A paragraph of statistical analysis has been added to the revised manuscript (lines 224-230, unmarked version).

We sincerely hope that the reviewer could approve our explanations.

<u>Responses to Reviewer 2</u>:

Comment 1: As mentioned in this paper the aim of this project was avoid the false negatives caused using the WHO-recommended primers and probes for detecting influenza A and B viruses, and to shorten total time needed to differentiate influenza A and B. Authors must be clarified this time under 2.5 hours, is include the time of viral RNA extraction? If is include, how many samples?

Response: We thank the reviewer for reminding us this important issue. The time of viral RNA extraction was not included in the test duration of 2.5 hours, which was indicated only for the multiplex RT-PCR assay. In response to the reviewer's comment, we have added a statement in the revised manuscript to clarify this issue (lines 370-371, unmarked version). The statement reads as; "The total test duration did not include the time of viral RNA extraction."

Comment 2: Considering that there are currently rapid one-step commercial kits and some papers, for detecting several respiratory viruses at the same time, authors must be comprised their methods with these commercial kits in the discussion section of the article. such as: A multiplex real-time RT-PCR for simultaneous detection of four most common avian respiratory viruses

Response: We thank the reviewer for providing the valuable reference, which has been added to our reference list (ref. 25). In response to this comment, we have added

a paragraph (lines 378-385, unmarked version) to discuss the methods of that work (ref. 25). The paragraph reads as: “A recent work [25] developed a multiplex real-time RT-PCR for the simultaneous detection of the four most common avian respiratory viruses: avian influenza virus, infectious bronchitis virus, Newcastle disease virus, and infectious laryngotracheitis virus. They used various primers and probes for these viruses, which were encompassed into one tube for simultaneous detections. Their approach was similar to ours except that our multiplex assay was designed for the simultaneous detection of Influenza B virus or pdm H1N1 and H3N2, the two most prevalent strains of Influenza A.”

We have also added statements (lines 385-388, unmarked version) to discuss the methods of certain commercial kits and an additional reference (ref. 26) was added to the list. These statements read as: “Certain commercial kits such as FilmArray multiplex PCR [26] apply nucleic acid amplification test-based methods to detect multiple respiratory pathogens in a single test. However, unlike the results of our multiplex assay reporting C_T values, the results of these commercial kits show only positive or negative diagnosis.”

We sincerely hope that the reviewer could approve our explanations.

KG Support Limited
www.kgsupport.com
info@kgsupport.com
Phone/Fax +852 3018 1216

REVIEW CERTIFICATION

DATE: SEPTEMBER 25, 2022

TO WHOM IT MAY CONCERN:

This memo certifies that Li-Kuang Chen, the author(s) of the below mentioned document had contracted our English language document review service for one (1) file, the particulars of which are listed below. The English review was conducted using a two-stage process, in which two editors reviewed the file, both of whom are native English speakers.

We therefore attest to the quality of the reviewed document.

KG Support Limited (Hong Kong)
www.kgsupport.com
info@kgsupport.com

Date of Review	DESCRIPTION (file name)
September 24, 2022	A highly efficient and accurate method of detecting and subtyping Influenza A pdm H1N1 and H3N2 viruses with newly emerging mutations in the matrix gene in Eastern Taiwan
