# Supplementary material for: A highly efficient and accurate method of detecting and subtyping Influenza A pdm H1N1 and H3N2 viruses with newly emerging mutations in the matrix gene in Eastern Taiwan
Source: PLoS One. 2023 Mar 23;18(3):e0283074. doi: 10.1371/journal.pone.0283074 (PMC10035893; doi:10.1371/journal.pone.0283074)
Supplement: Attachment — Submitted filename: Responses to Comments from Editor_R2.pdf [file pone.0283074.s006.pdf]

Responses to Editor (PONE-D-22-10495_R2)

We would like to thank the editor for their extensive assessment of our manuscript, and for important and helpful comments and suggestions. We have taken all the remarks into account, in a manner that is described in detail below together with our responses to comments. We have responded to all the editor's comments in a point-by-point fashion and have revised the manuscript accordingly. The revised portions are indicated by "Track Changes". An "unmarked" clean version of revised manuscript has also been provided. We think that, following these suggestions, our manuscript has gained in clarity and hope that the changes made will be considered satisfactory.

Responses to Editor's Comments:

Major Comments:

Comment 1: While not mentioned in the abstract and introduction, the authors did phylogenetic analysis of 94 samples (44 pdm H1N1 and 50 H3N2). I am at a lost on the intention of the phylogenetic analysis, which needs to be mentioned in abstracts and introduction to strengthen the rationale of the study.

Response: We thank the editor for this excellent suggestion. In response to the suggestion, we have added statements in the abstract and introduction to address this issue. The statements in the abstract read as:" Further, the phylogenetic analyses of our samples revealed that the characteristics of these viruses were different from those

reported previously using samples collected during 2012–2013” (lines 54-56). The statements in the introduction section read as:” In this study, we also report the results of the phylogenetic analyses of Influenza A pdm H1N1 and H3N2 viruses associated with the pandemic that occurred during 2012–2013 in Taiwan [14]. Since then, the changes in phylogenetic characteristics of these viruses have not yet been investigated. Therefore, in this study, we further performed phylogenetic analyses for the Influenza A pdm H1N1 and H3N2 viruses that caused the 2011–2015 pandemic” (lines 117-122).

Comment 2: In addition, the quality of the language still needs to be improved. We suggest you thoroughly copyedit your manuscript for language usage, spelling, and grammar. If you do not know anyone who can help you do this, you may wish to consider employing a professional scientific editing service.

Response: In response to the suggestion, our revised manuscript (R2 version) has been professionally edited by Editage for improvements of language; this company is recommended by the journal. The certificate of this English editing service has been uploaded alongside the revised manuscript.

Specific comments:

Comment 1: Abstract, line 45 – 46: This sentence is not relevant. Suggest deletion.

Response: In response to the suggestion, the sentence has been deleted (at the position

of line 45).

Comment 2: Materials and method, line 209: BLAST please reference "J Mol Biol. 1990 Oct 5;215(3):403-10. doi: 10.1016/S0022-2836(05)80360-2. Basic local alignment search tool S F Altschul 1, W Gish, W Miller, E W Myers, D J Lipman"

Response: We thank the editor for providing this important reference, which has been added to the reference list and cited (Materials and method, line 233). The numbers of other references in the text have been revised accordingly.

Comment 3: Results, line 247 – 248: Suggest moving tables 3 & 4 to supplemental materials.

Response: In response to the suggestion, these two tables have been moved to supplemental materials (S1 and S2 Tables) (line 273). The number of Table 5 in the text have been changed to Table 3 accordingly (line 274 and line 290).

Comment 4: The purpose of the 44 pdm H1N1 and 50 H3N2 samples for phylogenetic analysis.

Response: In response to this suggestion, we have added statements in the abstract section to address this issue." Further, the phylogenetic analyses of our samples revealed that the characteristics of these viruses were different from those reported previously using samples collected during 2012–2013" (lines 54-56). The statements in

the introduction section read as:” In this study, we also report the results of the phylogenetic analyses of Influenza A pdm H1N1 and H3N2 viruses associated with the pandemic that occurred during 2012–2013 in Taiwan [14]. Since then, the changes in phylogenetic characteristics of these viruses have not yet been investigated. Therefore, in this study, we further performed phylogenetic analyses for the Influenza A pdm H1N1 and H3N2 viruses that caused the 2011–2015 pandemic” (lines 117-122).

Comment 5: Discussion, line 341 – 344: This sentence is not relevant. Suggest deletion.

Response: In response to the suggestion, the sentence has been deleted (line 408).

Comment 6: Discussion, line 357 – 377: Suggest moving this paragraph to the beginning of the discussion.

Response: We thank the editor for the suggestion. This paragraph has been moved to the first paragraph of the discussion (lines 365-394).

Comment 7: Discussion, line 366 – 368: Please explain how the delayed CT values help distinguishing pdm H1N1 and H3N2. Where is Table 1A?

Response: Table 1A was a typo and should be Table 4 in our last R1 version. The Table 1A has been corrected to S2 Table (supplementary materials) in this R2 version. We apologize for the error we made. We have also added statements to explain how the delayed CT values help distinguishing pdm H1N1 and H3N2. These statements read

as:” In this study, the delayed CT values obtained when the MAF and MAR primers and MA probe were used (S2 Table) could be used to distinguish Influenza A pdm H1N1 and H3N2 serotypes. Specifically, Influenza A pdm H1N1 virus could be identified when the CT values obtained based on the use of the MAF and MAR primers and MA probe were very similar to those obtained when the InfAF and InfAR primers and InfA probe were used (S1 Table). Conversely, Influenza A H3N2 could only be identified when the CT values obtained based on the use of MAF and MAR primers and the MA probe were much greater than those detected based on the use of InfAF and InfAR primers and InfA probe or were even undetectable (S2 Table)” (lines 375-383).

editage

Editing Certificate

This document certifies that the manuscript listed below has been edited to ensure language and grammar accuracy and is error free in these aspects. The logical presentation of ideas and the structure of the paper were also checked during the editing process. The edit was performed by professional editors at Editage, a division of Cactus Communications. The author's core research ideas were not altered in any way during the editing process. The quality of the edit has been guaranteed, with the assumption that our suggested changes have been accepted and the text has not been further altered without the knowledge of our editors.

MANUSCRIPT TITLE

A highly efficient and accurate method of detecting and subtyping Influenza A pdm H1N1 and H3N2 viruses with newly emerging mutations in the matrix gene in Eastern Taiwan

AUTHORS

Hui-Hua Yang, I-Tsong Huang, Ren-Chieh Wu, Li-Kuang Chen

ISSUED ON

December 21, 2022

JOB CODE

FRGDS_1_2

THE EDITAGE Promise QUALITY GUARANTEE

Vikas Narang

Vikas Narang
Chief Operating Officer - Editage

editage

CACTUS

Editage, a brand of Cactus Communications, offers professional English language editing and publication support services to authors engaged in over 1300 areas of research. Through its community of experienced editors, which includes doctors, engineers, published scientists, and researchers with peer review experience, Editage has successfully helped authors get published in internationally reputed journals. Authors who work with Editage are guaranteed excellent language quality and timely delivery.

impact.science

researcher.life

lifesciences.cactusglobal.com

GLOBAL :
+1(833) 979-0061 | request@editage.com

TAIWAN :
0800668636 | submitjobs@editage.com
