# Supplementary material for: A highly efficient and accurate method of detecting and subtyping Influenza A pdm H1N1 and H3N2 viruses with newly emerging mutations in the matrix gene in Eastern Taiwan
Source: PLoS One. 2023 Mar 23;18(3):e0283074. doi: 10.1371/journal.pone.0283074 (PMC10035893; doi:10.1371/journal.pone.0283074)
Supplement: Attachment — Submitted filename: Responses to Comments from Editor_R3 .pdf [file pone.0283074.s007.pdf]

Responses to Editor (PONE-D-22-10495_R3)

We would like to thank the editor for their extensive assessment of our manuscript, and for important and helpful comments and suggestions. We have taken all the remarks into account, in a manner that is described in detail below together with our responses to comments. We have responded to all the editor's comments in a point-by-point fashion and have revised the manuscript accordingly. The revised portions are indicated by "Track Changes". An "unmarked" clean version of revised manuscript has also been provided. We think that, following these suggestions, our manuscript has gained in clarity and hope that the changes made will be considered satisfactory.

Responses to Editor's Comments:

General comment: The revised manuscript showed significant improvement over previous version. However, there are a few issues that still need to be addressed.

Response: We thank the editor for his/her positive feedback and for valuable suggestions to enhance the quality of our work. We also thank the editor for his/her careful reading of our manuscript.

Comment 1: Line 83, suggest changing "Therefore, presently,..." to "Presently..."

Response: This has been changed (Line 83).

Comment 2: Line 171, delete "of"

Response: This has been deleted (Line 171).

Comment 3: Line 268, suggest changing "Further, the..." to "The..."

Response: This has been changed (Line 268).

Comment 4: Line 268 - 269, It seems that table S1 and S2 only listed representative samples of pdmH1N1, and H3N2, not all the test samples. Please clarify.

Response: The editor is correct regarding this issue. Table S1 and S2 only listed representative samples of pdmH1N1, and H3N2, not all the test samples. In response to this suggestion, we have rephrased the statement as:" The threshold cycle (C_T) values of some representative tested samples diagnosed …" (Line 268-269). Additionally, the titles of tables S1 and S2 have been changed to "… detected from representative clinical samples…"(Supplemental Materials).

Comment 5: Line 271, suggest deleting "data"

Response: This has been deleted (Line 271).

Comment 6: Line 309 - 310, need to rephrase this sentence for clarity. Does the second set of primers and probe remedy the situation mentioned in the previous sentences?

Response: We thank the reviewer for reminding us this important issue. In response to the suggestion, the statement has been rephrased and reads as:" This situation can be remedied by our second set of primers and probe (InfAF and InfAR primers, and InfA

probe), which targeted a more conserved region of the M gene sequence of Influenza A H3N2 virus” (Line 309-311).

Comment 7: Line 369, suggest changing "...sometimes causes..." to "...caused by..."

Response: This has been changed (Line 369).

Comment 8: Line 370, suggest deleting "simultaneously applied"

Response: This has been deleted (Line 370).

Comment 9: Line 371-372, suggest deleting "...with early CT values..."

Response: This has been deleted (Line 372).

Comment 10: Line 419, suggest deleting "...previous..."

Response: This has been deleted (Line 419).

editage

Editing Certificate

This document certifies that the manuscript listed below has been edited to ensure language and grammar accuracy and is error free in these aspects. The logical presentation of ideas and the structure of the paper were also checked during the editing process. The edit was performed by professional editors at Editage, a division of Cactus Communications. The author's core research ideas were not altered in any way during the editing process. The quality of the edit has been guaranteed, with the assumption that our suggested changes have been accepted and the text has not been further altered without the knowledge of our editors.

MANUSCRIPT TITLE

A highly efficient and accurate method of detecting and subtyping Influenza A pdm H1N1 and H3N2 viruses with newly emerging mutations in the matrix gene in Eastern Taiwan

AUTHORS

Hui-Hua Yang, I-Tsong Huang, Ren-Chieh Wu, Li-Kuang Chen

ISSUED ON

December 21, 2022

JOB CODE

FRGDS_1_2

THE EDITAGE Promise QUALITY GUARANTEE

Vikas Narang

Vikas Narang
Chief Operating Officer - Editage

editage

Editage, a brand of Cactus Communications, offers professional English language editing and publication support services to authors engaged in over 1300 areas of research. Through its community of experienced editors, which includes doctors, engineers, published scientists, and researchers with peer review experience, Editage has successfully helped authors get published in internationally reputed journals. Authors who work with Editage are guaranteed excellent language quality and timely delivery.

GLOBAL :
+1(833) 979-0061 | request@editage.com

TAIWAN :
0800668636 | submitjobs@editage.com

CACTUS

impact.science

researcher.life

lifesciences.cactusglobal.com
