# Supplementary material for: A highly efficient and accurate method of detecting and subtyping Influenza A pdm H1N1 and H3N2 viruses with newly emerging mutations in the matrix gene in Eastern Taiwan
Source: PLoS One. 2023 Mar 23;18(3):e0283074. doi: 10.1371/journal.pone.0283074 (PMC10035893; doi:10.1371/journal.pone.0283074)
Supplement: Attachment — Submitted filename: PONE-D-22-10495(1).docx [file pone.0283074.s004.docx]

Comments to authors:

The manuscript entitled” A highly efficient and accurate method for detecting and subtyping Influenza A pdm H1N1 and H3N2 viruses with newly emerging mutations in the matrix gene in Eastern Taiwan” (Manuscript Number: PONE-D-22-10495) was reviewed, the number of samples were studied in this manuscript are very valuable. These results can provide information on clinically correct medication and vaccine design, but some cases not clear such as:

1) As mentioned in this paper the aim of this project was avoid the false negatives caused using the WHO-recommended primers and probes for detecting influenza A and B viruses, and to shorten total time needed to differentiate influenza A and B. Authors must be clarified this time under 2.5 hours, is include the time of viral RNA extraction? If is include, how many samples?
2) Considering that there are currently rapid one-step commercial kits and some papers, for detecting several respiratory viruses at the same time, authors must be comprised their methods with these commercial kits in the discussion section of the article.

Title: A multiplex real-time RT-PCR for simultaneous detection of four most common avian respiratory viruses

Dear editor:

The manuscript entitled” A highly efficient and accurate method for detecting and subtyping Influenza A pdm H1N1 and H3N2 viruses with newly emerging mutations in the matrix gene in Eastern Taiwan” (Manuscript Number: PONE-D-22-10495) was reviewed. The paper is not good enough to be published in Plos One; some major revision to be done:

1) As mentioned in this paper the aim of this project was avoid the false negatives caused using the WHO-recommended primers and probes for detecting influenza A and B viruses, and to shorten total time needed to differentiate influenza A and B. Authors must be clarified this time under 2.5 hours, is include the time of viral RNA extraction? If is include, how many samples?
2) Considering that there are currently rapid one-step commercial kits for detecting several respiratory viruses at the same time, authors must be comprised their methods with these commercial kits in the discussion section of the article.
3) There is not novelty in this manuscript.
